# A Systematic Review of CBM Content in Practitioner-Focused Journals: Do We Talk About Instructional Decision-Making?

**DOI:** 10.1177/00222194231215031

**Published:** 2023-12-07

**Authors:** Erica C. Fry, Jessica R. Toste, Beth R. Feuer, Christine A. Espin

**Affiliations:** 1 The University of Texas-Austin, USA; 2 Leiden University, The Netherlands

**Keywords:** curriculum-based measurement, data-based decision-making, content analysis

## Abstract

Data-based decision-making (DBDM) using curriculum-based measurement (CBM) data has demonstrated effectiveness in improving academic achievement for students with or at risk for learning disability. Despite substantial evidence supporting DBDM, its use is not common practice for many educators, even those who regularly collect CBM data. One explanation for its lack of widespread use is that educators may not receive adequate training in the DBDM aspects of CBM. Espin et al. examined the extent to which DBDM is represented in CBM professional development (PD) materials and found that the topic was significantly underrepresented (12% to 14% of CBM PD material content) compared with other CBM topics. The purpose of this study was to conduct a conceptual replication of the Espin et al. systematic review through an analysis of CBM content in practitioner journal articles. The present review includes 29 practitioner articles coded to the four CBM categories used in the Espin et al. study: (a) general CBM information, (b) conducting CBM, (c) data-based decision-making, and (d) other. Results revealed a pattern similar to the one found by Espin et al. with approximately 18% of the content of practitioner articles on CBM devoted to the topic of decision-making. These findings strengthen the recommendation from Espin et al. for increased attention to DBDM in CBM training materials.

For students with or at risk of learning disability, the use of progress monitoring data is essential for timely evaluation of the effectiveness of academic interventions and responsive, appropriate instructional decision-making. Federal special education legislation (Individuals with Disabilities Education Improvement Act [IDEA], 2004) and subsequent litigation (e.g., *Endrew F. v. Douglas County School District*, 2017) have established a clear mandate for educators to monitor, document, and respond to student progress. To do so, educators commonly collect curriculum-based measurement (CBM) data for progress monitoring and reporting purposes, but previous research indicates that they are far less likely to use CBM data to guide their instructional decision-making.

Data-based instruction (DBI), also referred to as data-based individualization (Fuchs et al., 2021; National Center on Intensive Intervention [NCII] at American Institutes for Research, 2013) or data-based program modification (Deno & Mirkin, 1977), is a process for adjusting interventions by responding to CBM data. The process involves implementation of an intervention program and ongoing progress monitoring with a valid and reliable system, such as CBM. When data indicate a student is not making adequate progress in response to the intervention, the next step is to use diagnostic assessment data to inform an adjustment to the intervention, with the goal of increasing the rate of student progress. After an adjustment is implemented, the process is repeated to continue monitoring student progress. Meta-analyses of DBI effectiveness support its use for improving academic performance across content areas (Jung et al., 2018; Kim & Choi, 2021).

One critical component of the DBI process is data-based decision-making (DBDM), using student data to directly inform individual instructional decisions. Despite the demonstrated effectiveness of using CBM data for DBDM, many educators lack the knowledge and skills needed to engage in this type of instructional decision-making (e.g., Espin et al., 2018; Oslund et al., 2021; Stecker et al., 2005; van den Bosch et al., 2017). Fortunately, when educators receive CBM training that addresses components of DBDM, their knowledge and skills related to data use may improve (Filderman et al., 2021; Gesel et al., 2021; Wayman & Jimerson, 2014). Research has demonstrated positive effects when training focuses on essential CBM content—collecting, graphing, and interpreting CBM data as well as linking data to instruction (i.e., DBDM; McMaster et al., 2020; van den Bosch et al., 2017, 2019). What remains unclear is the extent to which this CBM content, particularly DBDM, is explicitly taught in educator preparation programs and professional development (PD) training opportunities.

## What Is CBM?

In the late 1970s and 1980s, Stanley Deno and a team of researchers at the University of Minnesota worked to develop an assessment system that would allow educators to quickly interpret student performance data and identify when there is a need for instructional adjustment (Deno, 1985). The goal was not simply to quantify and report student performance, but rather to use assessment as a tool to directly inform instruction. They aimed to develop a system that produced reliable and valid scores, could be easily understood, and was simple, efficient, and inexpensive to administer. Deno and his colleagues developed a system that not only met these criteria but also demonstrated effectiveness as a tool that educators were able to use to effect greater student achievement (Deno et al., 1982; Deno & Fuchs, 1987; Fuchs & Deno, 1991, 1992).

The system incorporates parallel forms of measures that are aligned with the curriculum and are of equal difficulty to allow for detection of small changes in performance over time. Since this early work, CBM in reading has been established as a standardized, technically adequate, formative assessment system with reliability ranging from .82 to .99 (e.g., Keller-Margulis et al., 2008; Marston, 1989; Reschly et al., 2009). Furthermore, reading CBM data are strongly predictive of overall reading performance and aligned with standardized test scores (e.g., Espin et al., 2008; Fuchs et al., 1988; Wayman et al., 2007). In fact, scores from CBM reading measures have been found to be accurate for screening and identifying children as young as first grade as at risk of failing standardized reading assessments in later grades (Hintze & Silberglitt, 2005).

While early CBM research focused primarily on reading, measures have since been developed across content and skill areas. Reported reliability and validity estimates of CBM assessment vary somewhat across these areas. For example, in mathematics, CBM measuring number sense has demonstrated reliability ranging from .78 to .99 and validity ranging from .49 to .93 (Clarke & Shinn, 2004). In the elementary grade levels, mathematics computation CBM have demonstrated reliability and validity above .90 (e.g., Keller-Margulis et al., 2008; Marston, 1989). Mean criterion validity of .55 has been reported in meta-analyses of writing CBM assessments (Romig et al., 2017). Finally, a meta-analysis of science and social studies CBM found significant heterogeneity among studies and alternate form reliability between .21 and .89 (Conoyer et al., 2022). Although variability exists across content and skill areas, there is evidence that CBM is an effective option for collecting academic progress data and informing instruction (Busch & Espin, 2003; Gansle et al., 2006; VanDerHeyden & Burns, 2005).

For the purposes of DBI, CBM data are graphed and visually analyzed. Each CBM probe produces a single datapoint, a snapshot of student performance at a specific moment in time. Once baseline data are collected, a goal is developed using one of multiple possible strategies that take into account factors such as performance at baseline, length of the intervention, achievement norms, and anticipated rate of growth (Fuchs & Fuchs, 2002; Hosp et al., 2016). The next step is to plot baseline data and the long-range achievement goal on a graph where the x-axis displays time and the y-axis displays scores. A line drawn from baseline to the goal serves as an aim line (sometimes referred to as a goal line), modeling the expected performance at each data collection point if the student is to reach the goal according to the predetermined timeline. As data are plotted over time, a trendline may be added to show the student’s predicted trajectory based on all the data collected so far.

Essential knowledge and skills are required not only to administer, collect, graph, and interpret CBM data but also to engage in instructional decision-making in response to that data. There are two key decisions that educators make while collecting CBM progress data: *when* there is a need to adjust instruction and, following from that decision, *how* to adjust instruction (NCII at American Institutes for Research, 2013). To determine when an instructional adjustment is needed, educators engage in visual analysis of CBM graphs and apply decision rules (e.g., Van Norman & Christ, 2016). How does the slope of the trendline compare to the slope of the aim line? At the intervention’s projected end date, is the trendline above, below, or similar to the aim line? How much variability exists within the plot of data points? Do the data vary consistently about the aim line, or do multiple consecutive points fall either above or below the line? If there is a need to adjust instruction, educators must consider how to do so in a way that meets the student’s needs (Fuchs et al., 2021). There is likely a need to consider additional diagnostic data to supplement the CBM progress data (Powell et al., 2022). For example, if the CBM graph displays three consecutive data points falling below the projected aim line, the educator may determine that the intervention is not adequately meeting the student’s needs and it is time to adjust instruction. To determine how to do this, the educator may review their anecdotal behavior notes and decide to collect antecedent-behavior-consequence (ABC) data to better understand how they can intensify instruction for this student. Taken together, the educator may decide to incorporate a 60-s movement break during the 30-min intervention. They will implement this change, while continuing to collect and graph CBM progress data to monitor the student’s response over time .

## Educators’ Use of CBM for Instructional Decision-Making

CBM is commonly used for tracking and reporting student progress toward annual goals on Individualized Education Programs (IEPs) and for universal screening to determine which students receive targeted or intensive instruction within a multi-tiered system of supports (MTSS). These uses of CBM are relatively straightforward—they generally make use of CBM data collected at a single timepoint and do not require expertise beyond the knowledge necessary for reliable administration and scoring. However, DBI requires a broader range of skills that support frequent, ongoing collection and interpretation of progress data for students who have the most persistent and severe academic difficulties.

To improve academic outcomes for students who require targeted or intensive intervention and support, practitioners must develop expertise in reading and interpreting CBM graphs for the purpose of making effective instructional decisions. Manualized CBM procedures generally provide guidance for graph interpretation, but educators may find themselves lacking the expertise to engage in this type of DBDM. For example, Wachen et al. (2018) noted that educators were generally unable to “bridge the divide between using data to identify students in need of help and using data to modify instruction” (p. 296). That is, educators have a wealth of high-quality academic achievement data and little clarity on what to do with it.

A consistent and worrisome finding to emerge from both general and special education research is that educators have difficulty using data to guide instructional decision-making (e.g., Espin et al., 2018; Mandinach & Gummer, 2013; van den Bosch et al., 2017). Although CBM graphs appear relatively uncomplicated, research on graph comprehension in general reveals that reading even simple graphs can be challenging (see Friel et al., 2001). Graph interpretation is subject to a variety of cognitive errors such as being influenced by prior knowledge, seeing relationships in the data that do not exist, and confusing slope with height (Glazer, 2011). To become proficient in the use of CBM for DBDM, educators must have access to high-quality training opportunities and materials.

## Professional Training Materials

Although some special educators receive CBM training in their educator preparation programs, many enter the profession without training in research-based practices (Brownell et al., 2010; Kennedy et al., 2016), including CBM. Educators whose preparation did not include training in CBM may subsequently receive in-service PD in CBM. Given that the ultimate purpose of collecting CBM progress data for students with and at risk for learning disability is to have valid and reliable data to guide *instructional decision-making*, one might reasonably assume that CBM training materials for special education educators would include substantial information on DBDM. Effective CBM implementation relies upon developing educators’ knowledge and skill in multiple areas; DBDM is arguably among the most critical for intensifying instruction.

In their 2021 systematic review, Espin and colleagues examined the proportion of attention devoted to DBDM compared with other aspects of CBM in PD training materials. They calculated the distribution of CBM information across four categories: general information, conducting CBM, DBDM, and other (e.g., CBM used for screening or RTI placement). Next, they compared these proportions to two alternate distributions: that which would be observed if the four categories were given equal attention and the distribution recommended by a panel of experienced CBM trainers. Espin et al. (2021) found that little emphasis was placed on DBDM relative to other CBM topics in the PD materials that they reviewed. The actual proportion (12% for presentations, 14% for manuals and books) was significantly less than would be expected from either an equal distribution (25%) or the proportion recommended by experienced trainers (34%). While these findings suggested a lack of training focused on DBDM, the authors noted a need to replicate the study with other materials wherein educators may access CBM content. The present review attempts to do just that.

To identify sources used by educators to gain information about best practices, the Education Week Research Center (2017) conducted a survey of more than 500 educators from a nationally representative K–12 sample. Educators reported that they most frequently relied on content from PD (78%), followed by word of mouth or recommendations from colleagues (71%), educator-focused websites (50%), and social media (40%). In addition, more than a quarter of respondents (26%) reported accessing information about best practices through practitioner-focused journals. A similar survey of more than 1,300 educators, conducted by the University of Virginia’s Jefferson Education Exchange, asked educators which channels they use to access research. At least 24% of respondents reported accessing information through a subscription to an academic journal (Friedman, 2018). These recent surveys suggest that academic journals represent a significant source of professional learning and are likely accessed by approximately one-quarter of U.S. educators. Considering that this is an outlet frequently used by special education researchers to translate research, there is a need to explore CBM content within practitioner-focused journals.

## Study Purpose

The purpose of the present systematic review was to conduct a conceptual replication of Espin and colleagues (2021). Specifically, we sought to extend their findings by examining the extent to which DBDM is addressed in CBM-focused articles written for a practitioner audience. While Espin et al. (2021) focused on professional development materials, this study focuses on information presented in practitioner-oriented journals. Many educators report obtaining information from journals, and although they also report getting information from other sources (e.g., recommendations from colleagues, websites, and social media), it is likely that the information from these other sources is also based in part on information published in journals. Because so many educators lack knowledge and skill in DBDM, it is important to understand the representation of CBM content across the various sources used to gather information.

Increasingly, there are investments being made in effective dissemination and knowledge translation efforts of educational research fundings (e.g., National Academies of Sciences, Engineering, and Medicine [NASEM], 2022). Writing for practitioner audiences remains a key outlet for such efforts; however, little is known about the content or quality of these resources. The present review contributes to our understanding of how current research dissemination efforts may support educators’ professional learning about using CBM data for DBDM. We anticipate the findings of the present review will reflect trends similar to those identified by Espin et al. (2021), although we do not present hypotheses due to the exploratory nature of this study. We sought to address three research questions:

**Research Question 1:** What proportion of information is devoted to DBDM, relative to other content categories, in CBM practitioner-focused articles?
**Research Question 2:** Does the proportion of information devoted to DBDM differ from what would be expected if information were equally distributed across major CBM content categories?
**Research Question 3:** When DBDM content is included, what is the specificity with which articles address when and how to make instructional decisions using CBM data?

## Method

### Search and Selection Process

**Figure 1.**
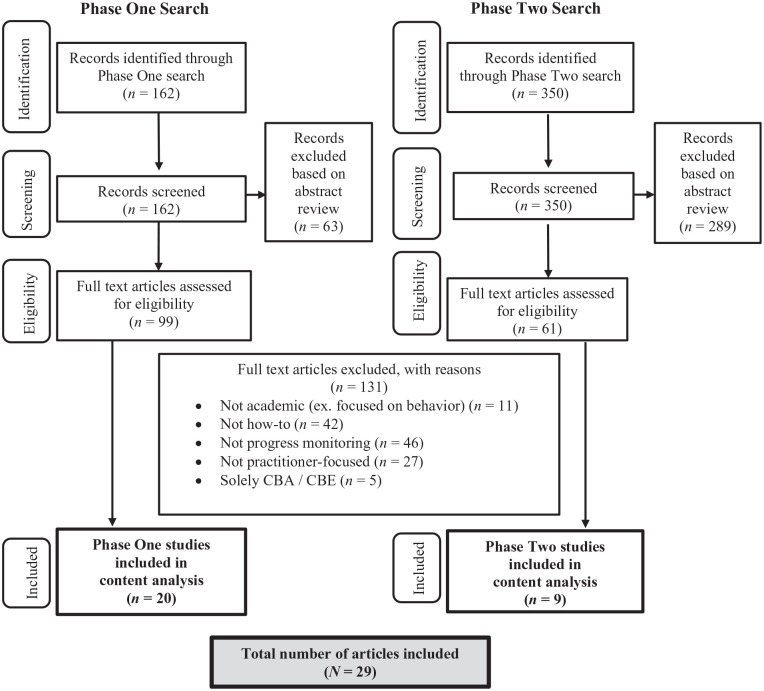
Search and Selection Process.

An overview of the search process is presented in Figure 1. We identified articles to be considered for inclusion through two phases of comprehensive journal searches—an initial search of four practitioner-focused journals, followed by an expanded search of eight additional journals based on expert recommendations. Each phase is described below.

To be eligible for inclusion in the study, articles had to meet the following criteria: (a) published in English, (b) written for a practitioner audience, (c) appeared in an indexed journal, (d) included a “how-to” component, and (e) focused on assessment of academic skills. Articles were deemed to be written for a practitioner audience if the primary purpose was to convey information educators could readily apply to their practice, not to report findings or discuss implications for practice derived from empirical research. Among these practitioner-focused articles, there must have also been a “how-to” component, which we operationalized as a description of how to administer CBM or use CBM data for the purpose of progress monitoring.

#### Phase 1: Initial Search of Practitioner-Focused Journals

This systematic review of peer-reviewed special education practitioner journals began with an electronic search of four special education practitioner journals: *Beyond Behavior, Intervention in School and Clinic*, *Learning Disabilities Research & Practice*, and *TEACHING Exceptional Children*. As widely distributed practitioner publications of the Council for Exceptional Children (CEC) and Council for Learning Disabilities (CLD), we anticipated that these were journals special educators would likely access for information on research-based practices, including CBM. Titles and abstracts of articles published between the journal’s inception and March 2022 were searched with the terms used by Espin et al. (2021): *curriculum-based measurement*, *CBM, general outcome measurement, data-based instruction, data-based decision-making, curriculum-based assessment*, and *curriculum-based evaluation*. In addition, we included the following terms to broaden the scope of the search and ensure that all relevant articles were retrieved: *data-based individualization*, *DBI*, *progress monitoring*, and *academic progress monitoring*. The Boolean operator “OR” was entered between each search term to capture all relevant results.

To ensure we captured all relevant articles, we conducted a comprehensive search of each journal’s publication history, including full-text searches via the publishers’ sites and database searches of titles, abstracts, and subject headings with each journal using Education Source and PsycINFO. After deduplication, this search process resulted in 162 articles. Through abstract screening, 63 were excluded for the following reasons: not practitioner-focused (*n* = 20), focused on progress monitoring of behavior rather than academics (*n* = 18), did not discuss any form of progress monitoring (*n* = 18), did not include a “how-to” component (*n* = 5), and focused solely on curriculum-based assessment to the exclusion of CBM (*n* = 2). In total, 99 articles were retrieved for full-text review.

#### Phase 2: Expanded Search Based on Expert Recommendations

Our selection of four practitioner journals in Phase 1 was based on the opinions of the research team—and perhaps an assumption that widely distributed journals are accessible to practitioners. Thus, to mitigate these potential limitations, we solicited input from published researchers in the field to conduct an expanded search (Phase 2). To identify additional journals to be searched for relevant articles, the first authors of all articles identified in Phase 1 were contacted. Authors were asked to complete a survey that briefly described the current review, listed the four journals already included, and asked for suggestions of additional journals that were peer-reviewed, indexed in electronic academic databases, and practitioner-focused where special educators would be likely to find information on CBM.

In total, 19 authors responded to the survey, collectively recommending an additional 11 journals. To determine whether each journal was peer-reviewed and indexed, we verified each title on ProQuest, through the University library stem, and their own publisher website. Of these journals, three were eliminated as they were not indexed or peer-reviewed, resulting in an additional eight journals: *Assessment for Effective Intervention*, *Contemporary School Psychology*, *Journal of Applied School Psychology*, *Preventing School Failure*, *Psychology in the Schools*, *Remedial and Special Education*, *School Psychology Forum*, and *The Reading Teacher*. These journals were searched using the same procedures described in Phase 1. This search returned 350 results published before March 2022. Of these, 289 were excluded for the following reasons: not practitioner-focused (*n* = 221), did not discuss any form of progress monitoring (*n* = 45), focused on progress monitoring of behavior rather than academics (*n* = 22), and focused solely on curriculum-based assessment (CBA) or curriculum-based evaluation (CBE; *n* = 1). Following the screening process, 61 articles were retrieved for full-text review.

#### Full-Text Review

Next, the 160 identified articles (99 from Phase 1 and 61 from Phase 2) were retrieved and reviewed for eligibility based on the established inclusion criteria. Through this process, 131 articles were excluded for the following reasons: did not discuss any form of progress monitoring (*n* = 46), did not include a ”how-to” component (*n* = 41), were not practitioner-focused (*n* = 28), focused on progress monitoring of behavior rather than academics (*n* = 11), and focused solely on CBA or CBE (*n* = 5). A total of 29 articles from 5 journals met criteria for inclusion in the present systematic review. For full-text review, two authors independently evaluated each of these 160 articles for inclusion. Reliability between researchers was 93.75%; calculated by dividing the number of articles with agreement (*n* = 150) by the total number of articles. All discrepancies were resolved through discussion to reach consensus.

### Coding Procedures

Prior to coding, we verified that none of the 29 eligible articles had Supplemental files available via the journal website or other service for hosting open materials. We then used Research Electronic Data Capture (REDCap; Harris et al., 2009, 2019) tools to extract data related to the general features of each article. This information included author(s), journal name, article title, year of publication, content area (e.g., reading, spelling, and math), type of CBM data (e.g., oral reading fluency, digits correct, correct word sequences), presence of a vignette (yes/no), presence of graph(s), content of graph (images only, discussion only, images and discussion of graph), and grade level(s) of focus (elementary, middle school, and high school). We also coded specificity of DBDM content, with each article being assigned two holistic codes: (a) *When* to make an instructional change, and (b) *How* to make an instructional change. In each category, DBDM content was coded as *none* if the article did not mention instructional decision-making, *reference* if instructional decision-making was mentioned but not described, *non-specific guidance* if types of instructional adjustments in response to student data were mentioned (e.g., increase dosage or raise the goal), and *specific guidance* if there was clear explanation for how educators should engage in a decision-making process in response to interpretation of CBM data to inform instructional adjustments. Two researchers independently coded all articles and reliability was calculated at 90%; all discrepancies were discussed until consensus was reached.

Next, plain text versions of all 29 articles were prepared and files were imported into NVivo12 for Mac. All text within each article was coded to identify CBM content using a protocol that replicated and extended Espin et al. (2021): (a) general CBM information, (b) conducting CBM, (c) CBM DBDM, (d) CBM other, (e) non-CBM assessment, and (f) other text. The inclusion of the category *other text* allowed every paragraph to be coded. Table 1 provides an operational definition, examples, and non-examples for each code.

**Table 1.** CBM Content Categories and Definitions.

Code	Description	Includes	Does not include
General CBM Information	General information, CBM background	Background / explanation of what CBM is; research base for CBM / reference to CBM studies	Information on non-CBM assessment
Conducting CBM	Administering CBM, collecting or graphing CBM data	Selecting, creating, administering, scoring CBM; graphing CBM data; goal setting	DBDM or other steps that occur after graphing
CBM DBDM	Reading or interpreting CBM graphs, linking data to instruction	Comparing slopes; comparing data points to goal; data-decision rules; clear link to instruction must be made	Decision-making that is not based on interpretation of graphed PM data
CBM Other	Using CBM for purposes beyond progress monitoring	Using data to screen and identify students with learning difficulties	Other forms of (non-PM) data used for screening / identification
Non-CBM Assessment	Content focused on other forms of assessment	CBA, CBE, benchmark exams, educator-made assessments, portfolios	Progress monitoring data
Other Text	Any text not captured in another code	Background information; organization/purpose of article	Assessment and collaboration

*Note.* CBM = curriculum-based measurement; DBDM = Data-based decision-making; CBA = curriculum-based assessment; CBE = curriculum-based evaluation; PM = progress monitoring.

Each paragraph of the article was assigned to only one of these six codes. Paragraphs were operationalized as chunks of text visually separated from one another by blank lines. Section headings were coded with the ensuing paragraph, and bulleted lists were coded with the preceding paragraph. Our unit of analysis differed from Espin et al. (2021), who coded an approximate proportion of a page (i.e., 1, 1/2, 1/3, 1/4) devoted to a topic, due to the nature of scholarly articles. One idea is rarely represented by a single sentence; as such, coding at the paragraph-level allowed for capturing the main ideas conveyed within each article. In articles containing a vignette or other narrative example, the content of the vignette was coded separately from the main text of the article to determine whether the proportion of text devoted to each category would vary according to the type of text. Again, two independent researchers completed this qualitative coding and reliability was calculated at 88%, with discrepancies discussed and consensus reached.

## Results

### Article Characteristics

In all, 29 articles met the eligibility criteria for inclusion in the present study (see Table 2). The articles were published between 1990 and 2022 across five journals: *Intervention in School and Clinic* (*n* = 9), *TEACHING Exceptional Children* (*n* = 9), *Preventing School Failure* (*n* = 6), *Assessment for Effective Intervention* (*n* = 3), and *Learning Disabilities Research and Practice* (*n* = 2). Twelve of the articles focused on reading CBM, seven focused on math, four focused on writing, one focused on social studies vocabulary, and the remaining five articles described CBM in multiple content areas. Twenty articles included vignettes, which were coded and analyzed separately from the main text of the article. Analyses required calculation of the total number of words in articles. The 29 articles included a total of 106,784 words—83,152 words in the main text and 23,632 words in the vignettes. The distribution of the total word count is displayed in Tables 3 and 4.

**Table 2.** Characteristics of the Articles from the Search and Selection Process.

Authors	Year	Journal	Content area and focus	Vignette	How	When
Busch & Espin	2003	AEI	Vocabulary Vocabulary matching as a measure of progress in content areas	Yes	Non-specific	Non-specific
Fuchs & Fuchs	1991	PSF	Mathematics Overview of applications of CBM	Yes	Specific	Non-specific
Fuchs et al.	2008	AEI	Mathematics Illustrates the systematic curricular sampling approach to formulating CBM systems	Yes	Non-specific	Specific
Gable & Hendrickson	1990	PSF	Reading Linking ORF error analysis to instruction	Yes	Reference	Reference
Hosp & Hosp	2003	PSF	Mathematics, Reading, Spelling Collecting CBM data	No	Reference	None
Marston et al.	1992	PSF	Reading Administering and interpreting CBM-R	No	Non-specific	Specific
Stecker et al.	2008	PSF	Reading CBM for instructional decision-making	Yes	Specific	Non-specific
Whinnery & Stecker	1992	PSF	Mathematics Four uses of CBM to enhance instructional decision-making	Yes	Non-specific	Non-specific
Yell & Stecker	2003	AEI	Reading CBM to develop compliant IEPs	Yes	Reference	Reference
Hasbrouck et al.	1999	LDRP	Reading Case studies from a CBM user	Yes	Specific	Specific
Groves Scott & Konya Weishaar	2003	ISC	Reading Step-by-step use of CBM at secondary level	No	Specific	Specific
Filderman et al.	2019	ISC	Reading DBDM at the secondary level	Yes	Non-specific	Specific
Dennis et al.	2014	ISC	Mathematics CBM with error analysis for skills analysis	Yes	Reference	Reference
Poch et al.	2022	ISC	Writing Steps of DBI to adjust writing instruction	Yes	Non-specific	Non-specific
McArthur Capizzi & Barton-Arwood	2009	ISC	Reading Graphic organizer worksheet as a guide for implementing CBM	Yes	Non-specific	Non-specific
Whinnery & Fuchs	1992	LDRP	Mathematics Computer application to link CBM data to instruction	Yes	Non-specific	Specific
Burns	2002	ISC	Mathematics, Reading Case studies of CBM use in general and special education settings	No	Specific	Specific
Fuchs et al.	2015	ISC	Mathematics, Reading, Spelling Overview of CBM and its implementation	No	Non-specific	Specific
Foegen & Morrison	2010	ISC	Mathematics Algebra CBM used in the classroom	No	Specific	Specific
Dombek & Al Otaiba	2015	ISC	Writing Administering and scoring K-2 writing CBM	No	Reference	Reference
McMaster et al.	2011	TEC	Writing CBM writing tasks for early identification and intervention	Yes	Specific	Non-specific
Bundock et al.	2018	TEC	Reading Reducing variability in ORF CBM data	Yes	Reference	Non-specific
Goo et al.	2012	TEC	Mathematics, Reading, Spelling, Writing Selecting web-based CBM system	Yes	Reference	Non-specific
Hessler & Konrad	2008	TEC	Writing CBM data for IEP writing and instruction	Yes	Specific	Specific
Pemberton	2003	TEC	Mathematics, Reading, Writing Using CBM to monitor and communicate academic progress	Yes	Reference	Reference
Garcia	2007	TEC	Reading Combining CBM and miscue analysis to inform instruction	No	Reference	None
Kretlow & Blatz	2011	TEC	Reading CBM for monitoring student progress	Yes	Non-specific	Non-specific
Foegen et al.	2016	TEC	Mathematics Selecting and using online algebra CBM	Yes	Non-specific	Non-specific
Filderman & Toste	2018	TEC	Reading Decision-making with CBM progress data	Yes	Reference	Specific

*Note.* AEI = *Assessment for Effective Intervention*; ISC = *Intervention in School and Clinic*; LDRP = *Learning Disabilities Research and Practice*; PSF = *Preventing School Failure*; TEC = *TEACHING Exceptional Children*; CBM = curriculum-based measurement; DBDM = Data-based decision-making; DBI = Data-based instruction; IEP = Individualized Education Programs; ORF = oral reading fluency.

**Table 3.** Distribution of CBM Content.

Source	General CBM	Conducting CBM	CBM DBDM	CBM Other	Non-CBM assessment	Other text	Total
Text							
# Articles	28	24	22	0	17	25	n/a
# Words	27,861	22,687	7,925	0	10,051	13,910	83,152
% Words	26.09%	21.25%	8.09 %	0%	9.41%	13.03%	77.87%
Vignette							
# Articles	13	18	15	2	5	11	n/a
# Words	4,506	7,477	5,571	748	878	4,452	23,632
% Words	4.22 %	7.00%	5.22%	0.70%	0.82%	4.17%	22.13%
Total	30.31%	28.25%	12.64%	0.70%	10.23%	17.2%	100%

*Note.* CBM = curriculum-based measurement; DBDM = Data-based decision-making.

**Table 4.** Distribution of CBM Content Across Four Major CBM Instructional Categories: Actual, Equal, and Recommended Distributions.

Source	General CBM	Conducting CBM	CBM DBDM	CBM Other	Total
Actual Distributions					
# Words	32,367	30,164	13,496	748	76,775
%Words	42.16%	39.29%	17.58%	0.97%	100%
Equal Distributions					
# Words	19,193	19,193	19,193	19,193	76,775
%Words	25%	25%	25%	25%	100
Experienced Trainers’ Recommendations^a^					
# Words	~13,435	~27,832	~25,911	~9,597	76,775
%Words	17.5%	36.25%	33.75%	12.50%	100%

*Note.* CBM = curriculum-based measurement; DBDM = Data-based decision-making.

a Number of words was calculated based on recommendations for distribution (%) of CBM content from experienced trainers, as reported in Espin et al. (2021).

Based on our final corpus of studies, we also calculated the relative representation of CBM content across the five practitioner-focused journals included in this review. These 29 articles represent approximately 0.35% of all articles published across all issues of these journals.

### Content in CBM Practitioner-Focused Articles

To address research question 1, the proportion of text coded to each of the six CBM content categories was calculated. Table 3 presents the percentage of words devoted to each category, separated according to whether the words appeared in the text of the article or the vignette. The category *General CBM Information* accounted for the greatest proportion of all text (30.31% or 32,367 words). Text coded to this category appeared in the main text of 28 articles and 13 vignettes. This category accounted for 26.09% of the main text (27,861 words), and 4.22% of vignette text (4,506 words) across all articles. The next largest percentage of text was coded to the *Conducting CBM* category (28.25%), followed by *Other Text* (17.2%), *CBM DBDM* (12.64%), *Non-CBM Assessment* (10.23%), and *CBM Other* (0.70%).

Next, we replicated the analysis reported by Espin et al. (2021). We examined the text coded to the four CBM categories originally used by those authors: *General CBM Information*, *Conducting CBM*, *CBM DBDM*, and *CBM Other*. In their analysis of PD materials, Espin and colleagues found that 54.7% of presentation slides and 49.6% of manual/book pages were devoted to conducting CBM; 24.5% of presentation slides and 21.9% of manual/book pages were devoted to general CBM information; 11.8% of presentation slides and 13.8% of manual/book pages were devoted to CBM DBDM; and 9.1% of presentation slides and 14.7% of manuals/books were devoted to CBM other. For the 29 practitioner-focused articles included in the present review, 42.2% of the text was devoted to *General CBM Information* and 39.3% to *Conducting CBM*. A substantially smaller proportion of text was devoted to *CBM DBDM* (17.6%), and a negligible proportion was devoted to *CBM Other* (1%; see Table 4).

### Distribution Across CBM Content Categories

Research question 2 focused on the distribution of text across the four main CBM content categories. Specifically, we examined the proportion of text devoted to the instructional decision-making aspect of CBM (i.e., DBDM) compared with the proportion that would be seen if the four categories were equally represented across these CBM-focused practitioner articles. As shown in Table 4, the observed proportion (17.6%) of information on DBDM was lower than the 25% one would expect if equal attention were to be devoted to each category. Espin et al. (2021) included an additional research question to further compare the observed distribution of professional development materials across the four categories to the distribution recommended by experienced CBM trainers, included in Table 4. While expert recommendations were specific to CBM content included in PD materials, it may reasonably be assumed that they indicate a desirable distribution of CBM content relevant to practitioner articles as well. According to these recommendations, the greatest emphasis should be placed on conducting CBM (36.8% of PD content) followed by CBM DBDM (33.8%), general CBM information (17.5%), and CBM other (12.5%).

### Specificity of DBDM Content

Finally, to address Research Question 3, we examined the specificity with which each article addressed the DBDM aspects of CBM. Unlike the process for exploring Research Questions 1 and 2, which quantified the proportion of text devoted to a particular topic, here we assigned holistic codes to each article. Specificity was coded based on the authors’ descriptions of *when* and *how* to make instructional decisions in response to CBM data. The following four codes were used to describe specificity: (1) *none*, (2) *reference*, (3) *nonspecific*, and (4) *specific*. We assigned specificity codes to each article based on two separate categories: (a) *When* to make an instructional change, and (b) *How* to make an instructional change. For example, a given article could be coded Reference for *when* to make a change and nonspecific for *how* to make a change. Of the articles that focused on DBDM content, 17.2% provided *specific* guidance regarding both when and how to make instructional adjustments, 20.7% provided *non-specific* information regarding both when and how to make instructional adjustments, and 17.2% included *references* to both when and how to make instructional adjustments. Another 10.5% of articles included a combination of references and nonspecific guidance while 27.5% included a combination of non-specific and specific guidance, and 6.7% included a reference to making instructional adjustments but no information regarding when to do so. Collectively, 31.0% of articles included specific guidance around either when or how to make an instructional change, but not both, while 51.8% of articles provided no specific guidance in either category. The degree of specificity present in an article was not associated with CBM content area, journal, or date of publication. Additional details and examples of the type of when and how descriptions included in the articles are provided in the following sections.

#### When to Make an Instructional Change

Articles were coded as references to *when* to make an instructional change if they mentioned making a decision but did not provide examples, descriptions, or guidance. They included statements such as: “Progress monitoring after instruction would then indicate if they are progressing at an adequate rate or if they are achieving below the aim. In the latter case, data would provide a guide for further instruction.” (Dombek & Al Otaiba, 2016, p. 282). Of the 29 articles included in the present review, 17% (*n* = 5) referenced when to make and instructional change.

Articles that provided non-specific guidance around *when* to make an instructional change mentioned making a decision in response to student data but did not offer details or descriptions of how practitioners might determine it was time to make a change. They included statements such as:

At regular intervals, the teacher applies standard decision-making rules to the graphed data and, when the student’s actual rate of progress is not as rapid as the anticipated rate of progress, the teacher implements an intervention likely to enhance student achievement (Yell & Stecker, 2003, p. 81).

Of the 29 articles, 38% (*n* = 11) provided nonspecific guidance on when to make a change.

Articles that provided specific guidance around *when* to make an instructional change included statements such as:

With this method, look at the last three data points collected versus the goal line. If a student is performing below the goal line for all three data points, an instructional change is appropriate. If a student is performing above the goal line for all three data points, a higher goal may be needed. If a student performs above and below the goal line, instruction may stay the same because the student is on target toward one’s goal (Filderman & Toste, 2018, p. 137).

Of the 29 articles, 35% (*n* = 10) provided specific guidance on when to make a change.

#### How to Make an Instructional Change

Articles that simply referenced *how* to make an instructional change mentioned the existence of decision-making but did not provide examples or descriptions of the types of adjustments that might be made. They included statements such as:

After the goal line is drawn on the graph, the teacher should enter CBM data on the graph each week in order to monitor and evaluate a student’s RTI. Graphing each data point as it is collected allows timely evaluation of RTI and needed instructional changes based on the data (McArthur Capizzi & Barton-Arwood, 2009, p. 20).

Of the 29 articles included in the present review, 35% (*n* = 10) referenced how to make a change. Articles that provided nonspecific guidance around *how* to make an instructional change mentioned categories of change such as goal, dosage, or scaffolding but did not offer details or descriptions. They included statements such as: “If a student is experiencing difficulty during the acquisition stage, manipulating variables such as the format of instruction or the difficulty level of the reading material may be appropriate” (Gable et al., 1990, p. 40). Of the 29 included articles, 38% (*n* = 11) provided nonspecific guidance on when to make and instructional change.

Articles that provided specific guidance around *how* to make an instructional change included statements such as:

Instructional changes can take several forms. The first type of change might be made to the environment in order to support the learners’ needs, such as changing the student’s seating during instruction to facilitate better attention or changing the time of instruction to better match the student’s learning preference. The second type of change involves modifications to the instructional design. You might change the type of instruction from small group to one-on-one, make sure the student has plenty of time for guided and independent practice of skills taught, provide reteaching when necessary, review skills, or simplify directions. Teachers might also look into changes that involve supplemental instruction. The student might need additional instruction in learning strategies, decoding skills, vocabulary, use of context clues, or visualization to be successful in the current curriculum. You might also want to do a student interview or “think aloud” during oral reading to determine what techniques the student is using during reading (Groves Scott & Konya Weishaar, 2003, p. 158).

Of the 29 articles, 27% (*n* = 8) provided nonspecific guidance on when to make a change.

## Discussion

Although CBM can be used for screening or benchmarking, arguably its most important feature is its sensitivity to change over time (Deno, 1985; Deno & Mirkin, 1977). For students with or at-risk of learning disability, CBM data can provide educators with critical information to intensify and individualize academic interventions—that is, to effectively implement DBI. When educators have the knowledge, skills, and awareness to respond appropriately to CBM data, they can minimize wasted instructional time and make changes to better align interventions with student learning needs (Fuchs et al., 2021; Kearns et al., 2021; Stecker et al., 2005).

### Does CBM Content Adequately Address DBDM?

The current review was initiated in response to the Espin et al. (2021) recommendation that their study be replicated with other CBM training materials, including articles in practitioner journals, to determine whether similar patterns would be observed. Thus, this study systematically reviewed articles on CBM from 12 practitioner-focused journals to examine the amount of attention devoted to DBDM. Journal articles represent an important source of information on conducting CBM that educators may access when they enter the profession. If they do not receive sufficient training in DBDM in their educator education programs, these practitioner articles may be a critical resource accessed by educators who are invested in their own professional growth and the success of their students. Recent survey data indicate that approximately one-quarter of U.S. educators access academic journals when looking for research to inform their selection of classroom practices and intervention strategies (Education Week Research Center, 2017; Friedman, 2018). Despite the importance of this topic, our comprehensive search of 12 journals resulted in only 29 articles (from 5 journals) focused on CBM content for a practitioner audience. This represents approximately 0.35% of all articles published across all issues of these journals.

The current review revealed that the proportion of materials dedicated to DBDM (i.e., the use of CBM for instructional decision-making) accounted for 17.58% of the content in practitioner-focused articles. This is a greater proportion than Espin et al. (2021), who reported that DBDM accounted for 13% of the content in CBM professional development materials. That said, this is still substantially less content than would be expected if equal attention were paid to each of the four CBM categories (25%) or if the recommendations of experienced trainers were followed (33.75%).

While, in their review of CBM PD materials, Espin et al. found the greatest proportion of attention was given to conducting CBM (53%) followed by general CBM information (24%), in the case of practitioner journal content, the greatest proportion of text was devoted to general CBM information (42.16%) followed by information on conducting CBM (39.29%). That is, most of the text in these articles focused on providing background and rationale for using CBM or the details of how to administer and collect CBM data. While general information on CBM and information on how to conduct CBM certainly is relevant, it is perhaps not where the most attention is needed. The knowledge and skills required to engage in effective DBDM are much more complex, and it is unclear where educators access training and instruction in this area. Another difference between the two reviews is that the *CBM Other* category made up more than 11% of content in Espin et al.’s review of PD materials, but less than 1% in the current review of practitioner-focused articles. It could be that CBM PD materials are geared toward a broader audience using CBM for a variety of purposes (e.g., school-wide screening, placement in RTI tiers for the delivery of focused or intensive intervention, and grouping for small group instruction) while special education practitioner journals may be more likely to focus specifically on CBM for progress monitoring.

### What Is the Specificity of DBDM-Focused Information?

In addition to quantitatively capturing the relative representation of various CBM topics in practitioner journal articles, a further aim of the present review was to describe the specificity with which DBDM was discussed. The purpose was to communicate not only how much coverage exists in the literature but also how *effectively* the DBDM content might equip educators to engage in instructional decision-making. Although 38% of the articles provided specific information describing how to determine *when* to make an instructional adjustment, less than half of that group (17% of all articles) also described specifically *how* to make those adjustments. At present, DBDM content within CBM practitioner articles is lacking in both the quantity and the specificity that would be required to equip educators to improve their data practices and impact student learning.

Research has demonstrated that many educators struggle to interpret CBM graphs and to connect data to instruction. Educators may be skilled in CBM administration procedures overall, though remain unsure of what to do with the data they collect. Fuchs and Fuchs (2002, 2021) examined the extent to which computers may be utilized for a variety of CBM tasks including scoring, graphing, and data analysis. These authors noted that educators consistently had difficulty connecting assessment data to instructional decisions, so they explored the effectiveness of computer-generated instructional recommendations. Although the recommendations were successful in helping teachers identify what students needed to be taught, they failed to identify student-level instructional adjustments. This complex skill falls to individual educators who need targeted training to gain proficiency in DBDM.

### Limitations

Although the present study reflects a comprehensive review of CBM content in practitioner-focused journals, it is constrained by several limitations. First, by choosing to analyze content from a specific set of journals, it is possible that some articles may have been missed. We attempted to limit this possibility by expanding the selection of included journals based on input from CBM researchers, ultimately considering inclusion from a total of 15 journals and systematically reviewing content in 12 of those journals. However, this may not account for all available content.

A second limitation is the focus on articles published in practitioner-focused journals. While these may be the most likely journals to which educators have access, it is also possible that they are relying on other, more easily accessible, resources when seeking information on CBM. Many educators are not members of the professional organizations that provide journal access, and it is reasonable to assume that free resources are more likely to be accessed than those requiring a subscription. These include materials shared on blogs or social media, and resources from sites such as *Teachers Pay Teachers* which include both free and low-cost paid content. Recent work has suggested that close to 70% of educators in the United States have downloaded at least one resource from TPT to use in their classrooms (Shelton et al., 2022).

### Directions for Future Research

An opportunity exists to review syllabi, slides, and textbooks from CBM and assessment courses in educator preparation programs to assess the amount of attention devoted to various CBM topics. Identifying gaps in content at the pre-service level may better inform PD design for those currently in the classroom as well as support efforts to strengthen the preparation of future educators. Further research should also be informed by input from educators to identify the resources they most often consult when seeking information on research-based practices. Content analyses of educator-identified resources may yield greater insights into the most common classroom practices as well as provide researchers with more impactful dissemination options.

Authors of future practitioner articles on CBM may consider including specific guidance for educators who are unsure how to link their CBM data to instruction and when to make instructional adjustments. Examples of this type of specific guidance can be found in several articles included in the present review (i.e., Burns, 2002; Foegen & Morrison, 2010; Groves Scott & Konya Weishaar, 2003; Hasbrouck et al., 1999; Hessler & Konrad, 2008). Additionally, future practitioner articles should move beyond how and why to conduct CBM. There is a need to provide guidance around interpreting student data and connecting that data to instructional planning and intensification. The relative dearth of information on DBDM within CBM practitioner articles is not an indication that current published articles are of poor quality; existing articles simply had alternate areas of focus within the topic of CBM. Rather, this gap in published content highlights an opportunity to build upon what has been published to extend the conversation to focus on what educators can do with the student data they collect.

As we move forward in this research, there is a need to examine what school leaders know about CBM, progress monitoring, and using data to make individual instructional decisions. This line of inquiry will support a more comprehensive understanding of the reasons educators make instructional decisions that are not grounded in data. Although educators enjoy some degree of autonomy within their classrooms, their ability to select interventions and assessments and to engage in specific data-use practices are constrained by a variety of factors including district policies, school culture, and the knowledge and priorities of school leaders. Without the support of school leadership teams, educators may not be afforded the opportunity to engage in research-based practices such as DBDM. It is worth considering the ways in which the training and development of educational leaders create school contexts that either hinder or promote educators’ ability to apply their pedagogical knowledge and skills.

### Conclusion

Knowledge of DBDM is essential for effective DBI implementation and meeting the needs of students with or at-risk of learning disability, especially those who require intensive interventions. Knowing the proportion of attention devoted to DBDM within practitioner articles relative to other CBM topics is of little value on its own. The meaningful contribution of the present study is that it demonstrates what is missing when educators turn to practitioner articles to learn about CBM. Although educators are likely to learn a great deal about what CBM is and how it is administered, they are far less likely to learn about the most important and impactful aspects of CBM, the steps that come after the data are graphed.

When a student is not making adequate progress, the data point not to a deficit in the child, but rather to a failure of the intervention as currently delivered to support them in reaching the learning goal. Shifting the focus of collecting CBM data from quantifying and reporting student performance to informing instructional practice is a necessary precursor to providing the most effective intervention. Few practices are supported by an evidence base as robust as the one that has been established for using CBM data to make instructional decisions, but students don’t benefit from the evidence until it informs the instructional practices in their classrooms. Additional work is needed to translate CBM research into materials that are accessible and widely available. If effective dissemination strategies are implemented, DBDM has the potential to improve classroom practices and drive student achievement. Until educators are skilled in graph reading, data interpretation, and instructional decision-making, their use of CBM will not adequately support student learning and achievement.

## References

[bibr1-00222194231215031] Brownell M. T. Sindelar P. T. Kiely M. T. Danielson L. C. (2010). Special education teacher quality and preparation: Exposing foundations, constructing a new model. Exceptional Children, 76(3), 357–377. 10.1177/001440291007600307

[bibr2-00222194231215031] *Bundock K. O’Keeffe B. V. Stokes K. Kladis K. (2018). Strategies for minimizing variability in progress monitoring of oral reading fluency. TEACHING Exceptional Children, 50(5), 273–281. 10.1177/0040059918764097

[bibr3-00222194231215031] *Burns M. K. (2002). Comprehensive system of assessment to intervention using curriculum-based assessments. Intervention in School and Clinic, 38(1), 8–13. 10.1177/1053451202038001020

[bibr4-00222194231215031] *Busch T. W. Espin C. A. (2003). Using curriculum-based measurement to prevent failure and assess learning in the content areas. Assessment for Effective Intervention, 28(3–4), 49–58. 10.1177/073724770302800306

[bibr5-00222194231215031] Clarke B. Shinn M. R. (2004). A Preliminary Investigation Into the Identification and Development of Early Mathematics Curriculum-Based Measurement. School Psychology Review, 33(2), 234–248.

[bibr6-00222194231215031] Conoyer S. J. Therrien W. J. White K. K. (2022). Meta-analysis of validity and review of alternate form reliability and slope for curriculum-based measurement in science and social studies. Assessment for Effective Intervention, 47(2), 101–111. 10.1177/1534508420978457

[bibr7-00222194231215031] *Dennis M. S. Calhoon M. B. Olson C. L. Williams C. (2014). Using computation curriculum-based measurement probes for error pattern analysis. Intervention in School and Clinic, 49(5), 281–289. 10.1177/1053451213513957

[bibr8-00222194231215031] Deno S. L. (1985). Curriculum-based measurement: The emerging alternative. Exceptional Children, 52(3), 219–232. 10.1177/001440298505200303 2934262

[bibr9-00222194231215031] Deno S. L. Fuchs L. S. (1987). Developing curriculum-based measurement systems for data-based special education problem solving. Focus on Exceptional Children, 19(8), 1–16. 10.17161/foec.v19i8.7497

[bibr10-00222194231215031] Deno S. L. Mirkin P. K. Chiang B. (1982). Identifying valid measures of reading. Exceptional Children, 49, 36–45. 10.1177/001440298204900105 7140791

[bibr11-00222194231215031] Deno S. L. Mirkin P. K. (1977). Data-based program modification: A manual. Council for Exceptional Children.

[bibr12-00222194231215031] *Dombek J. L. Al Otaiba S. A. (2016). Curriculum-based measurement for beginning writers (K–2). Intervention in School and Clinic, 51(5), 276–283. 10.1177/1053451215606691

[bibr13-00222194231215031] Education Week Research Center. (2017). Data: Where do teachers get their ideas? https://www.edweek.org/teaching-learning/data-where-do-teachers-get-their-ideas

[bibr14-00222194231215031] Endrew, F. v. Douglas County School District, 580 U.S. (2017).

[bibr15-00222194231215031] Espin C. A. Wallace T. Campbell H. Lembke E. S. Long J. D. Ticha R. (2008). Curriculum-based measurement in writing: Predicting the success of high-school students on state standards tests. Exceptional Children, 74(2), 174–193. 10.1177/001440290807400203

[bibr16-00222194231215031] Espin C. A. Saab N. Pat-El R. Boender P. D. van der Veen J. (2018). Curriculum-based measurement progress data: Effects of graph pattern on ease of interpretation. Zeitschrift für Erziehungswissenschaft [Journal of Pedagogical Science], 21(4), 767–792. 10.1007/s11618-018-0836-9 30956566 PMC6428331

[bibr17-00222194231215031] Espin C. A. van den Bosch R. M. van der Liende M. Rippe R. C. Beutick M. Langa A. Mol S. E. (2021). A systematic review of CBM professional development materials: Are teachers receiving sufficient instruction in data-based decision-making? Journal of Learning Disabilities, 54(4), 256–268. 10.1177/0022219421997103 33749351

[bibr18-00222194231215031] *Filderman M. J. Austin C. R. Toste J. R. (2019). Data-based decision making for struggling readers in the secondary grades. Intervention in School and Clinic, 55(1), 3–12. 10.1177/1053451219832991

[bibr19-00222194231215031] *Filderman M. J. Toste J. R. (2018). Decisions, decisions, decisions: Using data to make instructional decisions for struggling readers. TEACHING Exceptional Children, 50(3), 130–140. 10.1177/0040059917740701

[bibr20-00222194231215031] Filderman M. J. Toste J. R. Didion L. Peng P. (2021). Data literacy training for K–12 teachers: A meta-analysis of the effects on teacher outcomes. Remedial and Special Education, 43(5), 328–343. 10.1177/07419325211054208

[bibr21-00222194231215031] *Foegen A. Morrison C. (2010). Putting algebra progress monitoring into practice: Insights from the field. Intervention in School and Clinic, 46(2), 95–103. 10.1177/1053451210375302

[bibr22-00222194231215031] *Foegen A. Stecker P. M. Genareo V. R. Lyons R. Olson J. R. Simpson A. Romig J. E. Jones R. (2016). Using an online tool for learning about and implementing algebra progress monitoring. TEACHING Exceptional Children, 49(2), 106–114. 10.1177/0040059916674327

[bibr23-00222194231215031] Friedman S. (2018, November 27). How educators utilize research. THE Journal. https://thejournal.com/articles/2018/11/27/how-educators-utilize-research.aspx

[bibr24-00222194231215031] Friel S. N. Curcio F. R. Bright G. W. (2001). Making sense of graphs: Critical factors influencing comprehension and instructional implications. Journal for Research in Mathematics Education, 32(2), 124–158. 10.2307/749671

[bibr25-00222194231215031] Fuchs L. S. Deno S. L. (1991). Paradigmatic distinctions between instructionally relevant measurement models. Exceptional Children, 57, 488–501. 10.1177/001440299105700603

[bibr26-00222194231215031] Fuchs L. S. Deno S. L. (1992). Effects of curriculum within curriculum-based measurement. Exceptional Children, 58, 232–243. 10.1177/001440299105800306 1813311

[bibr27-00222194231215031] *Fuchs L. S. Fuchs D. (1991). Curriculum-based measurements: Current applications and future directions. Preventing School Failure: Alternative Education for Children and Youth, 35(3), 6–11. 10.1080/1045988X.1991.10871068

[bibr28-00222194231215031] Fuchs L. S. Fuchs D. (2002). Computer applications to curriculum-based measurement. Special Services in the Schools, 17(1–2), 1–14. 10.1300/J008v17n01_01

[bibr29-00222194231215031] *Fuchs L. S. Fuchs D. Hamlett C. L. (2015). Curriculum-based measurement: A standardized, long-term goal approach to monitoring student progress. Intervention in School and Clinic, 50(3), 185–192. 10.1177/105345129002500508

[bibr30-00222194231215031] Fuchs L. S. Fuchs D. Hamlett C. L. Stecker P. M. (2021). Bringing data-based individualization to scale: A call for the next-generation technology of teacher supports. Journal of Learning Disabilities, 54(5), 319–333. 10.1177/0022219420950654 33813936

[bibr31-00222194231215031] Fuchs L. S. Fuchs D. Maxwell L. (1988). The validity of informal reading comprehension measures. Remedial and Special Education, 9(2), 20–28. 10.1177/074193258800900206

[bibr32-00222194231215031] *Fuchs L. S. Fuchs D. Zumeta R. O. (2008). A curricular-sampling approach to progress monitoring: Mathematics concepts and applications. Assessment for Effective Intervention, 33(4), 225–233. 10.1177/1534508407313484

[bibr33-00222194231215031] *Gable R. A. Hendrickson J. M. Meeks J. W. Evans S. S. Evans W. H. (1990). Curriculum-based measurement of oral reading: Linking assessment and instruction. Preventing School Failure: Alternative Education for Children and Youth, 35(1), 37–42. 10.1080/1045988X.1990.9944248

[bibr34-00222194231215031] Gansle K. A. VanDerHeyden A. M. Noell G. H. Resetar J. L. Williams K. L. (2006). The technical adequacy of curriculum-based and rating-based measures of written expression for elementary school students. School Psychology Review, 35(3), 435–450.

[bibr35-00222194231215031] *Garcia T. (2007). Facilitating the reading process: A combination approach. TEACHING Exceptional Children, 39(3), 12–17. 10.1177/004005990703900302

[bibr36-00222194231215031] Gesel S. A. LeJeune L. M. Chow J. C. Sinclair A. C. Lemons C. J. (2021). A meta-analysis of the impact of professional development on teachers’ knowledge, skill, and self-efficacy in data-based decision-making. Journal of Learning Disabilities, 54(4), 269–283. 10.1177/0022219420970196 33203294

[bibr37-00222194231215031] Glazer N. (2011). Challenges with graph interpretation: A review of the literature. Studies in science education, 47(2), 183–210.

[bibr38-00222194231215031] *Goo M. Watt S. Park Y. Hosp J. (2012). A guide to choosing web-based curriculum-based measurements for the classroom. TEACHING Exceptional Children, 45(2), 34–40. https://journals.sagepub.com/doi/pdf/10.1177/004005991204500204

[bibr39-00222194231215031] *Groves Scott V. Konya Weishaar M. (2003). Curriculum-based measurement for reading progress. Intervention in School and Clinic, 38(3), 153–159. 10.1177/10534512030380030401

[bibr40-00222194231215031] Harris P. A. Taylor R. Minor B. L. Elliott V. Fernandez M. O’Neal L. McLeod L. Delacqua G. Delacqua F. Kirby J. Duda S. N. , & REDCap Consortium. (2019). The REDCap consortium: Building an international community of software platform partners. Journal of Biomedical Informatics, 95, 1–10. 10.1016/j.jbi.2019.103208 PMC7254481 31078660

[bibr41-00222194231215031] Harris P. A. Taylor R. Thielke R. Payne J. Gonzalez N. Conde J. G. (2009). Research electronic data capture (REDCap)—a metadata-driven methodology and workflow process for providing translational research informatics support. Journal of Biomedical Informatics, 42(2), 377–381. 10.1016/j.jbi.2008.08.010 18929686 PMC2700030

[bibr42-00222194231215031] *Hasbrouck J. E. Woldbeck T. Ihnot C. Parker R. I. (1999). One teacher’s use of curriculum-based measurement: A changed opinion. Learning Disabilities Research & Practice, 14(2), 118–126. 10.1207/sldrp1402_5

[bibr43-00222194231215031] *Hessler T. Konrad M. (2008). Using curriculum-based measurement to drive IEPs and instruction in written expression. TEACHING Exceptional Children, 41(2), 28–37. 10.1177/004005990804100204

[bibr44-00222194231215031] Hintze J. M. Silberglitt B. (2005). A longitudinal examination of the diagnostic accuracy and predictive validity of R-CBM and high-stakes testing. School Psychology Review, 34, 372–386. 10.1080/02796015.2005.12086292

[bibr45-00222194231215031] *Hosp M. K. Hosp J. L. (2003). Curriculum-based measurement for reading, spelling, and math: How to do it and why. Preventing School Failure: Alternative Education for Children and Youth, 48(3), 10–17. 10.1080/1045988X.2003.10871074

[bibr46-00222194231215031] Hosp M. K. Hosp J. L. Howell K. W. (2016). The ABCs of CBM: *A practical guide to curriculum-based measurement*. Guilford Publications.

[bibr47-00222194231215031] Individuals with Disabilities Education Act, 20 U.S.C. § 1400 (2004).

[bibr48-00222194231215031] Jung P. G. McMaster K. L. Kunkel A. K. Shin J. Stecker P. M. (2018). Effects of data-based individualization for students with intensive learning needs: A meta-analysis. Learning Disabilities Research & Practice, 33(3), 144–155. 10.1111/ldrp.12172

[bibr49-00222194231215031] Kearns D. M. Feinberg N. J. Anderson L. J. (2021). Implementation of data-based decision-making: Linking research from the special series to practice. Journal of Learning Disabilities, 54(5), 365–372. 10.1177/00222194211032403 34374574

[bibr50-00222194231215031] Keller-Margulis M. A. Shapiro E. S. Hintze J. M. (2008). Long-term diagnostic accuracy of curriculum-based measures in reading and mathematics. School Psychology Review, 37(3), 374–390.

[bibr51-00222194231215031] Kennedy M. J. Wagner D. Stegall J. Lembke E. Miciak J. Alves K. D. Brown T. Driver M. K. Hirsch S. E. (2016). Using content acquisition podcasts to improve teacher candidate knowledge of curriculum-based measurement. Exceptional Children, 82(3), 303–320. 10.1177/0014402915615885

[bibr52-00222194231215031] Kim D. Choi S. (2021). The effects of data-based instruction (DBI) for students with learning difficulties in Korea: A single-subject meta-analysis. PLOS ONE, 16(12), 1–23. 10.1371/journal.pone.0261120 PMC8699614 34941909

[bibr53-00222194231215031] *Kretlow A. G. Blatz S. L. (2011). The ABCs of evidence-based practice for teachers. TEACHING Exceptional Children, 43(5), 8–19. 10.1177/004005991104300501

[bibr54-00222194231215031] Mandinach E. B. Gummer E. S. (2013). A systemic view of implementing data literacy in educator preparation. Educational Researcher, 42(1), 30–37. 10.3102/0013189X12459803

[bibr55-00222194231215031] Marston D. B. (1989). A curriculum-based measurement approach to assessing academic performance: What it is and why do it. In Shinn M. R. (Ed.), Curriculum-based measurement: Assessing special children (pp. 18–78). The Guilford Press.

[bibr56-00222194231215031] *Marston D. Diment K. Allen D. Allen L. (1992). Monitoring pupil progress in reading. Preventing School Failure: Alternative Education for Children and Youth, 36(2), 21–25. 10.1080/1045988X.1992.9944265

[bibr57-00222194231215031] *McArthur Capizzi A. Barton-Arwood S. M. (2009). Using a curriculum-based measurement graphic organizer to facilitate collaboration in reading. Intervention in School and Clinic, 45(1), 14–23. 10.1177/1053451209338394

[bibr58-00222194231215031] *McMaster K. L. Du X. Parker D. C. Pinto V. (2011). Using curriculum-based measurement for struggling beginning writers. TEACHING Exceptional Children, 44(2), 26–34. 10.1177/004005991104400203

[bibr59-00222194231215031] McMaster K. L. Lembke E. S. Shin J. Poch A. L. Smith R. A. Jung P. G. Allen A. A. Wagner K. (2020). Supporting teachers’ use of data-based instruction to improve students’ early writing skills. Journal of Educational Psychology, 112(1), 1–21. 10.1037/edu0000358

[bibr60-00222194231215031] National Academies of Sciences, Engineering, and Medicine. (2022). The future of education research at IES: Advancing an equity-oriented science. 10.17226/26428

[bibr61-00222194231215031] National Center on Intensive Intervention at American Institutes for Research. (2013). Data-based individualization: A framework for intensive intervention. ERIC Clearinghouse.

[bibr62-00222194231215031] Oslund E. L. Elleman A. M. Wallace K. (2021). Factors related to data-based decision-making: Examining experience, professional development, and the mediating effect of confidence on teacher graph literacy. Journal of Learning Disabilities, 54(4), 243–255. 10.1177/0022219420972187 33185149

[bibr63-00222194231215031] *Pemberton J. B. (2003). Communicating academic progress as an integral part of assessment. TEACHING Exceptional Children, 35(4), 16–20. 10.1177/004005990303500403

[bibr64-00222194231215031] *Poch A. L. Allen A. A. Jung P. G. Lembke E. S. McMaster K. L. (2022). Using data-based instruction to support struggling elementary writers. Intervention in School and Clinic, 57(3), 147–155. 10.1177/10534512211014835

[bibr65-00222194231215031] Powell S. R. Bos S. E. King S. G. Ketterlin-Geller L. Lembke E. S. (2022). Using the data-based individualization framework in math intervention. TEACHING Exceptional Children, 20(10), 1–11. 10.1177/00400599221111114

[bibr66-00222194231215031] Reschly A. L. Busch T. W. Betts J. Deno S. L. Long J. D. (2009). Curriculum-based measurement oral reading as an indicator of reading achievement: A meta-analysis of the correlational evidence. Journal of School Psychology, 47(6), 427–469. 10.1016/j.jsp.2009.07.001 19808123

[bibr67-00222194231215031] Romig J. E. Therrien W. J. Lloyd J. W. (2017). Meta-analysis of criterion validity for curriculum-based measurementin written language. The Journal of Special Education, 51(2), 72–82. 10.1177/0022466916670637

[bibr68-00222194231215031] Shelton C. C. Koehler M. J. Greenhalgh S. P. Carpenter J. P. (2022). Lifting the veil on TeachersPayTeachers.com: An investigation of educational marketplace offerings and downloads. Learning, Media and Technology, 47(2), 268–287. 10.1080/17439884.2021.1961148

[bibr69-00222194231215031] Stecker P. M. Fuchs L. S. Fuchs D. (2005). Using curriculum-based measurement to improve student achievement: Review of research. Psychology in the Schools, 42(8), 795–819.

[bibr70-00222194231215031] *Stecker P. M. Lembke E. S. Foegen A. (2008). Using progress-monitoring data to improve instructional decision making. Preventing School Failure: Alternative Education for Children and Youth, 52, 48–58. 10.3200/PSFL.52.2.48-58

[bibr71-00222194231215031] van den Bosch R. M. Espin C. A. Chung S. Saab N. (2017). Data-based decision-making: Teachers’ comprehension of curriculum-based measurement progress-monitoring graphs. Learning Disabilities Research & Practice, 32(1), 46–60. 10.1111/ldrp.12122 PMC7433367 31221014

[bibr72-00222194231215031] van den Bosch R. M. Espin C. A. Pat-El R. J. Saab N. (2019). Improving teachers’ comprehension of curriculum-based measurement progress-monitoring graphs. Journal of Learning Disabilities, 52(5), 413–427. 10.1111/ldrp.12122 31221014 PMC7433367

[bibr73-00222194231215031] VanDerHeyden A. M. Burns M. K. (2005). Using curriculum-based assessment and curriculum-based measurement to guide elementary mathematics instruction: Effect on individual and group accountability scores. Assessment for Effective Intervention, 30(3), 15–31. 10.1177/073724770503000302

[bibr74-00222194231215031] Van Norman E. R. Christ T. J. (2016). How accurate are interpretations of curriculum-based measurement progress monitoring data? Visual analysis versus decision rules. Journal of School Psychology, 58, 41–55. 10.1016/j.jsp.2016.07.003 27586069

[bibr75-00222194231215031] Wachen J. Harrison C. Cohen-Vogel L. (2018). Data use as instructional reform: Exploring educators’ reports of classroom practice. Leadership and Policy in Schools, 17(2), 296–325.

[bibr76-00222194231215031] Wayman M. M. Wallace T. Wiley H. I. Tichá R. Espin C. A. (2007). Literature synthesis on curriculum-based measurement in reading. The Journal of Special Education, 41(2),85–120.

[bibr77-00222194231215031] Wayman J. C. Jimerson J. B. (2014). Teacher needs for data-related professional learning. Studies in Educational Evaluation, 42, 25–34. 10.1016/j.stueduc.2013.11.001

[bibr78-00222194231215031] *Whinnery K. W. Fuchs L. S. (1992). Implementing effective teaching strategies with learning disabled students through curriculum-based measurement. Learning Disabilities Research & Practice, 7(1), 25–32.

[bibr79-00222194231215031] *Whinnery K. W. Stecker P. M. (1992). Individual progress monitoring to enhance instructional programs in mathematics. Preventing School Failure: Alternative Education for Children and Youth, 36(2), 26–29. 10.1080/1045988X.1992.9944266

[bibr80-00222194231215031] *Yell M. L. Stecker P. M. (2003). Developing legally correct and educationally meaningful IEPs using curriculum-based measurement. Assessment for Effective Intervention, 28(3–4), 73–88. 10.1177/073724770302800308

